# The novel HLA‐A*24 allele, HLA‐A*24:487, identified in three unrelated bone marrow donors in Colombia

**DOI:** 10.1111/tan.14432

**Published:** 2021-10-12

**Authors:** David Guillermo Hernández, Nathalie Camacho Ramírez, Melissa Mosquera Martínez, Paola Andrea Cendales, Bernardo Armando Camacho

**Affiliations:** ^1^ Colombian Bone Marrow Donors Registry Instituto Distrital de Ciencia, Biotecnología e Innovación en Salud – IDCBIS Bogotá Colombia

**Keywords:** bone marrow, HLA, new allele, next‐generation sequencing

## Abstract

Identification of the novel HLA‐A*24:487 allele, which differs from HLA‐A*24:02:01:01 at one position.

The HLA Class I genes, HLA‐A, ‐B, and ‐C, are located within the region that forms the human major histocompatibility complex on the short arm of Chromosome 6 (6p21.3). The HLA genes are characterized by their hyperpolymorphism and actively participate in the outcome of the transplantation of hematopoietic progenitors.^1^ In the last version of the IPD‐IMGT/HLA Database (Version 3.45.0, 2021‐07‐12), a total of 6921 alleles have been assigned for the HLA‐A gene,^2^ which indicates its extreme polymorphism. We report here the identification of the HLA‐A*24:487 allele in three unrelated Colombian bone marrow donors, using next‐generation sequencing.

The high‐resolution HLA typing for the identification of the new allele was performed by Histogenetics® for the five HLA Loci (HLA‐A, ‐B, ‐C, ‐DRB1, ‐DDB1), using the technique described previously by Cereb et al.^3^ A total of 1280 blood samples were processed by the laboratory, all within the framework of the research project led by the Instituto Distrital de Ciencia, Biotecnología e Innovación en Salud (IDCBIS) entitled “*Estudios técnicos para el establecimiento y organización de un Registro Nacional de Donantes de Células Progenitoras Hematopoyéticas en Colombia*,” which includes in one of its objectives the creation of the first registry of HLA typed donors in the country. The complete typing results of the three individuals were: (1) A*24:487, 26:01:01; B*35:43:01, 49:01:01G; C*01:02:01G, 15:04:01; DRB1*08:02:01G, 11:01:01G; DQB1*03:01:01G, 04:02:01G; (2) A*02:01:01, 24:487; B*14:02:01G, 35:43:01; C*01:02:01G, 08:02:01G; DRB1*01:02:01G, 08:02:01G; DQB1*04:02:01G, 05:01:01G; and finally, (3) A*03:02:01, 24:487; B*35:43:01, 44:02:01G; C*01:02:01G, 16:04:01G; DRB1*04:02:01, 08:02:01G; DQB1*03:02:01G, 04:02:01G.

The novel HLA‐A*24:487 allele differs from its closest allele HLA‐A*24:02:01:01, by a single nucleotide change of adenine (A) to thymine (T) in codon 220 within exon 4 of the HLA‐A gene. This change A > T generates a non‐synonymous substitution of the codon, encoding the amino acid Valine (V) instead of Aspartic Acid (D) (Figure 1). The nucleotide sequence of the new A*24:487 allele was submitted to GenBank with accession numbers (MN907091, MN907092, and MT364234) and the name HLA‐A*24:487 have been assigned by the WHO Nomenclature Committee for Factors of the HLA System in February 2020.^4^

**FIGURE 1:**
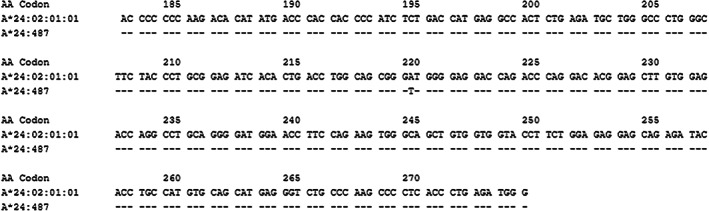
Alignment of the exon 4 sequence of the new HLA‐A*24:487 allele with the closest allele sequence HLA‐A*24:02:01:01. Identity with the sequence of the HLA‐A*24:02:01:01 allele is indicated with dashes. The number above the sequence corresponds to the position of the codon

These new findings will be useful since the alleles identified will provide an opportunity to improve the transplantation of hematopoietic progenitors, increasing the genetic diversity available for conducting searches for potential donors.^5^ Although the Latin American population does not have a significant representation in the largest registries in the world, through this type of report the need to continue identifying the genetic sequences of the population is noted,^6^ intending to be able to offer a treatment opportunity to all those patients who are currently waiting for a donation.

## CONFLICT OF INTEREST

The authors declare no potential conflict of interest.

## AUTHOR CONTRIBUTION

David Guillermo Hernández, Nathalie Camacho Ramírez, Melissa Mosquera Martínez, and Paola Andrea Cendales analysis and interpretation of results; writing and review of the final article also editing and structuring of the final document; Bernardo Armando Camacho supervised the project.

## Data Availability

The data that support the findings of this study are openly available in the IPD IMGT/HLA Database at https://www.ebi.ac.uk/cgi-bin/ipd/imgt/hla/get_allele.cgi?A*24:487.
